# Roles of Brassinosteroids in Mitigating Heat Stress Damage in Cereal Crops

**DOI:** 10.3390/ijms22052706

**Published:** 2021-03-08

**Authors:** Aishwarya Kothari, Jennifer Lachowiec

**Affiliations:** Plant Sciences and Plant Pathology Department, Montana State University, Bozeman, MT 59717, USA; aishwaryakothari@montana.edu

**Keywords:** plant hormones, thermal stress, agriculture, hormone transport, plants, development

## Abstract

Heat stress causes huge losses in the yield of cereal crops. Temperature influences the rate of plant metabolic and developmental processes that ultimately determine the production of grains, with high temperatures causing a reduction in grain yield and quality. To ensure continued food security, the tolerance of high temperature is rapidly becoming necessary. Brassinosteroids (BR) are a class of plant hormones that impact tolerance to various biotic and abiotic stresses and regulate cereal growth and fertility. Fine-tuning the action of BR has the potential to increase cereals’ tolerance and acclimation to heat stress and maintain yields. Mechanistically, exogenous applications of BR protect yields through amplifying responses to heat stress and rescuing the expression of growth promoters. Varied BR compounds and differential signaling mechanisms across cereals point to a diversity of mechanisms that can be leveraged to mitigate heat stress. Further, hormone transport and BR interaction with other molecules in plants may be critical to utilizing BR as protective agrochemicals against heat stress. Understanding the interplay between heat stress responses, growth processes and hormone signaling may lead us to a comprehensive dogma of how to tune BR application for optimizing cereal growth under challenging environments in the field.

## 1. Introduction

Threats to crop yields due to high temperatures require strategic interventions to maintain and increase productivity. Plant hormones underlie the “Green Revolution” of the mid-twentieth century, during which dwarfed crop varieties with altered hormone signaling were developed, providing massive increases in productivity of cereals [1]. Despite these successes, demand for food is rising quickly and outpacing projections of cereal production, requiring a surge in yields [2]. Cereal production is further imperiled by climate change, which threatens wheat, rice and maize yields worldwide [3]. Controlled environment studies have established that for every one degree increase in temperature above 15 °C during kernel development, a 3–5% reduction in grain yield of wheat occurs [4]. In the case of rice, grain yield decreases by 10% for every 1 °C increase in temperature during the growing season [5]. Correspondingly, studies from the field have reported up to 21% reduction in wheat yields due to heat and drought between 1980 and 2015 [6]. In order to combat the growing threat of high temperatures and meet food demand, producers require new crop varieties and improved management. A potential solution is to further leverage the success of the Green Revolution by fine-tuning the impact of plant hormones. Among plant hormones, brassinosteroids (BR) are polyhydroxylated steroids regulating a wide range of processes including plant growth and development [7,8]. Unlike auxins, gibberellins and abscisic acid, BR and their broad roles in plant development are often underrecognized, though known for nearly 50 years [9,10]. BR impact plant growth through promotion of cell elongation, cell division and differentiation [7,11] and increase fertility by enhancing pollen germination and pollen tube formation [12]. Indeed, a subset of Green Revolution varieties have altered BR action and show reduced stature and enhanced yields in cereals [13,14,15,16]. Along with promoting development, BR also protect plants from a range of abiotic and biotic stresses by modifying the mechanisms through which plants respond to stress. Hence, BR may represent an excellent target for manipulation to improve cereal resiliency to increasing temperatures. The scope of this review is to investigate how heat stress in cereals is connected to BR signal transduction and transport, while considering the use of exogenously applied BR to improve agronomic traits under high temperatures.

## 2. Diversity in BR Signaling

Brassinosteroids are found ubiquitously in the plant kingdom [11,17,18], though each species shows a unique complexity in BR action throughout development. Plant species have diverged in the metabolism and recognition of BR, each producing a suite of BR compounds with 81 natural BR identified so far [19]. Within a single species, BR are present variably in leaves, shoots, roots, pollen, fruits, seeds and vascular tissue [17], though pollen grains and immature seeds tend to have higher concentrations of BR than vegetative tissues relative to other plant hormones [20,21]. Further, different tissues throughout a plant may contain different BR compounds, and those concentrations can shift as plants age. For instance, in wheat, young developing tissues have higher concentrations of the BR 2,4-epibrassinolide than mature tissues [22]. These endogenous BR levels are regulated by biosynthesis and metabolism, well described in detailed reviews by Fujioka Yokota [23] and Wei Li [24], integrating feedback regulation triggered by BR signaling.

The diversity of BR compounds and concentrations are integrated into BR signal transduction to elicit developmental responses. The receptor kinase BRASSINOSTEROID INSENSITIVE 1 (BRI1) perceives BR at the cell surface [25], which leads to phosphorylation of BRI1 KINASE INHIBITOR 1 (BKI1), a negative regulator of BR. The inactivation of BKI1 allows BRI1 to heterodimerize with co-receptors, including BRI1-ASSOCIATED RECEPTOR KINASE 1 (BAK1) [26]. BRI1 and BAK1 transphosphorylate each other [27], leading to phosphorylation of BRASSINOSTEROID SIGNALING KINASE 1 (BSK1). BSK1 is released from the receptor complex and inactivates BRI1 SUPPRESSOR 1 (BSU1). BSU1 dephosphorylates and thus inactivates the BRASSINOSTEROID INSENSITIVE 2 kinase (BIN2) [28]. Inactivity of BIN2 leads to dephosphorylation of BRI1 EMS SUPPRESSOR 1 (BES1) and BRASSINAZOLE RESISTANT 1 (BZR1), allowing them to accumulate in the nucleus [29,30]. BES1 and BZR1 are TFs that bind to *cis*-regulatory regions of BR target genes and regulate physiology and development [31,32]. BES1 and BZR1 integrate signaling pathways through forming heterodimers with other TFs [31,33], relying on stress-sensitive HEAT SHOCK PROTEIN 90 [34,35], and mediating the crosstalk between BR and other hormones by directly binding to promoters of hormone synthesis genes [36].

Though the BR pathway just described is well-defined within *Arabidopsis thaliana*, there may exist additional modes of action in cereals. For example, diversity in BR signaling has been observed in rice. Studies of rice orthologs of AtBRI1, AtBZR1, AtBAK1 and AtBIN2 indicate that they are critical to BR signaling and have conserved function across species. On the other hand, rice-specific BR components LEAF AND TILLER ANGLE INCREASED CONTROLLER (LIC), DWARF and LOW-TILLERING (DLT) and TAIHU DWARF (TUD1) also act in BR signaling [37]. OsLIC is a TF that controls leaf and tiller angle and works antagonistically to OsBZR1 [38,39]. OsDLT positively regulates BR responses and controls plant height and tillering [40]. OsTUD1 acts epistatic to genes expressing G-proteins involved in BR signaling to mediate signaling [41]. In addition to varied components in BR signaling across species, the wide range of BR synthesized and differences in their specificity or activity may explain the observed variability in physiological effects of BR across research studies. However, the common feature of BR signaling regardless of mode is the output that BR alter gene expression [42].

## 3. BR Transport Is a Key to Understand Exogenous BR Action

Unlike other phytohormones, the effects of endogenous BR are local. Endogenous BR are synthesized at the site of action and not subjected to long-distance transport, instead regulated through the tissue specific control of BR synthesis, catabolism, and inactivation [43]. Possible molecular-level modes of BR export and short-distance transport are discussed in the review paper by Vukasinovic and Russinova [44]: BR can be actively transported by plasma membrane-localized transport, freely diffused through the plasma membrane, or diffused through plasmodesmata.

In contrast to endogenous BR, exogenously applied BR impact plants beyond the site and timing of application. The transport of exogenously applied BR in a plant depends on which species and organs BR was applied, though similar modes of transport have been observed in domesticated monocots and dicots. When applied to the roots, BR is transported rapidly to the leaves in cucumber and wheat. When applied to the third leaf of cucumber plants, BR was found to be transported to the fourth leaf at least by 7 days. Transport of the hormone was slower in wheat compared to cucumber in both applications. In addition, the cellular composition of plant organs appears to affect BR uptake and transport with BR was more easily taken up from the adaxial surface than the abaxial surface of cucumber leaves, but this was not seen in wheat [45]. Yokota, et al. [46] demonstrated that 6 h after applying BR to roots of rice seedlings, BR was taken up through the roots and translocated to shoots. When applied to rice shoots, BR was absorbed by the leaves, but only partially, while most of it remained on the leaf surface. Twenty-four hours after shoot treatment, most of the BR was found in the treated leaves, while only a little (about one-fourth) was found in the roots after 72 h [46]. This suggests that BR transport is slower from leaves to roots. Hence, when exogenously applied to leaves, BR is relatively immobile or moves very slowly. In contrast, when applied exogenously to roots, BR transport is faster and reaches the leaves, and likely other aerial tissues, implying that BR is transported through the xylem acropetally [47]. These results suggest that foliar application of BR may have minimal whole plant impacts, and other modes of application, such as seed application or irrigation, would be preferred.

The systemic impacts of exogenous BR may also be indirect, elicited instead through the induction of alternative hormone pathways through crosstalk [47]. Crosstalk between BR and other hormones causes changes in expression of genes involved in development and stress responses [48,49]. In *A. thaliana*, many auxin-responsive genes are also target genes of AtBES1 and AtBZR1 [50,51]. BR regulate gene expression of gibberellic acid metabolism genes to promote cell elongation in rice [52]. Li, et al. [53] showed that BR stimulate polar auxin transport modifying endogenous auxin levels by differentially regulating transcription of *PIN* gene family members in *A. thaliana*. Hormone crosstalk is also essential in strengthening the ability of plants to tolerate stress by controlling the balance between growth promotion and inhibition under unfavorable conditions [54]. The deficiency in BR transport studies indicate that more work needs to be done to characterize the transport pathway, crosstalk, and its importance in plant growth and development and heat stress tolerance for cereals.

## 4. BR Protect Plants under Heat Stress through Dual Roles

**Figure 1:**
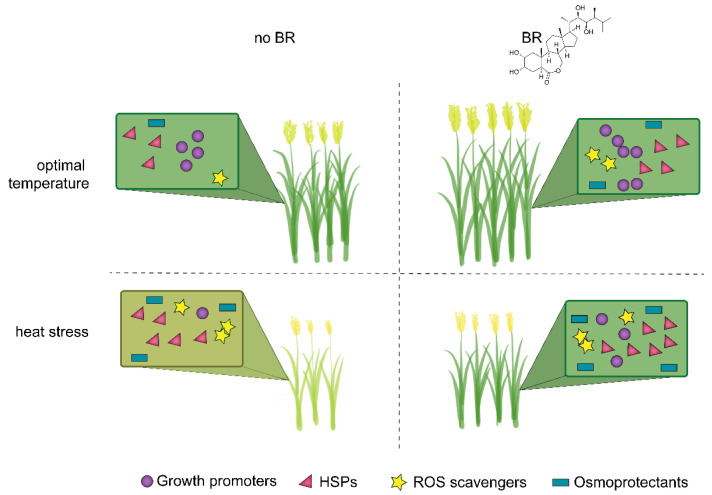
BR provide heat tolerance via compensation and priming of gene. Under optimal growth conditions, BR increase biomass and thereby yields by regulating the expression of growth promoters (like CESA, cyclins, other phytohormones). Under heat stress, BR compensate expression of growth and regulatory genes along with upregulating production of stress-responsive genes, proteins, and/or molecules to combat heat stress. Hence, even under heat stress, the plants have more biomass when treated with BR unlike untreated plants, almost similar to plants in optimal conditions.

Regardless of mode of transport, plants exposed to heat stress and exogenous BR show two major patterns of gene regulation: (1) BR rescue expression of developmental proteins that are suppressed under heat stress and (2) BR induce higher levels of protective proteins than heat stress alone (Figure 1). Transcriptomic analyses find that heat stress causes downregulation of many genes critical to cell wall synthesis, photosynthesis carbon assimilatory process, starch transport and accumulation, and many metabolic pathways [55,56]. Conversely, BR up-regulated genes are associated with plant growth and development processes, targeting genes encoding cell elongation and cell wall modification enzymes, auxin responsive factors, and TFs, among others [57,58,59], suggesting the mechanisms by which BR act to mitigate against heat stress.

Among the roles of BR via transcriptional impacts, their effects on biomass may be the most useful to exploit to improve crop yields and resiliency. Increasing grain yield is tightly correlated with increasing biomass [60]. Biomass is dependent on cell expansion and cell proliferation, both of which are regulated through BR signaling. BR induce plant growth via cell elongation by directly targeting expression of *CELLULOSE SYNTHASE* (*CESA*) genes in *A. thaliana,* thereby increasing cellulose content and biomass accumulation [61]. Heat stress inhibits cell elongation and causes cell cycle arrest through downregulation of genes such as *CESA* and certain cyclins [62,63]. Along with *CESA*, BR also stimulate the expression of cell wall expansion and loosening enzymes like expansins, xyloglucan endotransglucocylase, and pectin-lyase like [64,65]. Given that cell expansion is sensitive to heat stress in a manner that can be rescued by BR [66], possibly through targeting *CESA* and its homologs, BR can be used to increase crop biomass and thereby overall yield in both optimal and stressful conditions.

BRs’ impacts on cell cycle and cell division likely maintain biomass under heat stress as well, especially considering root biomass. The root quiescent cell center (QC) is located in the root meristematic region and is responsible for maintaining undifferentiated stem cells [67,68]. Heat stress can lead to DNA damage which then causes the QC to lose quiescence and enter active cell division phase to replace damaged cells [69,70]. BR regulate the cell cycle and proliferation of QC [71], activating QC division under stressful conditions [72,73]. During this process, BR represses the negative regulator of QC, BRASSINOSTEROIDS AT VASCULAR AND ORGANIZING CENTER (BRAVO) [72] and simultaneously targets the TF *ETHYLENE RESPONSE FACTOR 115* (*ERF115*) to promote QC division [74]. Speculation leads us to consider that the regulation of cell division by BR can be a way of tolerating heat stress.

The variety of heat stresses affecting cereals induce varied responses that can be mitigated though exogenous application of BR that primes the expression of protective molecules. Heat stresses vary in intensity, frequency, and duration. Corresponding to the characteristics of heat stress, plants have evolved specific responses to survive. Heat acclimation, also referred to as acquired thermotolerance, is the ability of plants to survive extreme temperatures after pre-conditioning to mild high temperatures (reviewed in [75,76]). For example, acclimation to high temperature during pre-anthesis stage in wheat has shown to alleviate the negative effects of high temperature during anthesis and post-anthesis stage increased yields as compared to non-acclimated plants [77]. However, these inherent mechanisms sustain only to a limit; beyond that, heat can have negative effects on plant growth and development resulting in fatal plant injuries and yield losses. Increasing the ability of plants to endure heat stress is critical in maintaining crop yields, especially in the face of climate change. Reinforcing evolved mechanisms to withstand heat stress may be achieved by leveraging BR.

Heat stress alters plant proteomic profiles to mitigate damage for survival. Most notable is the increased levels of heat shock proteins (HSPs). HSPs assist in the proper folding, intracellular distribution, and degradation of proteins, during stress as well as optimal conditions [78,79]. Consequently, HSPs provide heat tolerance by stabilizing proteins essential to physiological processes like photosynthesis, water use efficiency, and membrane stability [80,81,82]. In contrast to the upregulation of HSPs, a generalized repression of translation is seen due to heat stress, including genes like transporters, detoxifying enzymes and regulatory proteins to focus plant resources on combatting heat stress [55,56,83,84]. BR increase the expression of several classes of HSP, pointing to a protective role in heat tolerance. Indicating an essential role of BR in proper HSP induction, wild type barley varieties exhibit an increase in HSPs when plants were acclimated to high temperature, whereas BR mutant varieties show a reduced increase [85]. A class of HSPs, the small HSPs (sHSPs) are induced under heat stress and have a role in acclimation [86,87]. In barley, Sadura and colleagues confirmed that sHSPs accumulated in plants after heat acclimation [85]. sHSPs with functions in specific organelles protect essential cellular processes from heat stress. Evidence shows that accumulation of mitochondrial sHSPs provide heat acclimation by stabilizing electron transport chain complexes [88] and protect translational machinery during heat stress [89]. BRs amplify this protective response, with the application of exogenous BR increasing the accumulation of mitochondrial sHSPs in response to high temperature and provide thermotolerance as seen in tomato seedlings relative to seedlings not treated with BR [90]. In addition to mitochondria, chloroplasts require protection from heat stress, crucial for crops to continue to produce needed biomass for yields. Heat stress reduces plant photosynthetic capacity by damaging chloroplasts and photosynthetic machinery within [91,92]. Counteracting this, chloroplastic sHSPs are induced by heat stress and have shown to protect the photosynthetic machinery. In wheat, chloroplastic sHSPS are expressed in all parts of the plant including the flag leaf, immature and mature spikes, anther, carpel, and developing seeds [93]. Given that BR are known to rescue the rate of photosynthesis under high temperatures, and exogenous BR induce mitochondrial sHSPs, it will be valuable to inspect if BR also promote the accumulation of chloroplastic sHSPs. Studies using exogenous BR and examining the role of endogenous BR both support a major role in inducing the production of HSPs to help plants acclimate to heat stress.

The induction of osmoprotectants also alleviates the impacts of high temperatures, with BR acting to promote expression of these critical compounds. At the cellular level, heat stress destabilizes cellular membranes, which accelerates membrane injury and electrolyte leakage, thus leading to cell death and senescence [94]. Osmoprotectants such as betaines, sugars, and amino acids including free proline, maintain osmotic balance of cells under many unfavorable environmental conditions [95]. The action of osmoprotectants in response to heat stress have been studied across plant species for many abiotic stresses, but minimal work has been completed in cereals in response to heat stress. In rice, heat stress induces a sharp increase in the osmoprotectant γ-Aminobutyric acid (GABA) [96]. This increase in GABA was associated with reduced membrane injury and increased production of antioxidants, which improved survival of heat stressed plants. Though not yet examined in cereals, BR further increase the production of osmolytes under heat stress in other plants [97]. Consistent with these observations, BR application does protect cell membranes of wheat leaves under heat stress, reflected in the preservation of cell ultrastructure [98,99]. Though not yet determined directly, BR likely act to promote osmoprotectants under heat stress in cereals.

Heat stress is accompanied by oxidative stress as observed by the burst of hydrogen peroxide in cells after exposure to high temperature [100,101]. When plants are subjected to oxidative stresses, production of reactive oxygen species (ROS) is triggered. Among the cellular and molecular structures that ROS harm, oxidative damage to chloroplasts and the photosynthetic apparatus decrease plant photosynthetic capacity [102], reducing yield. To combat ROS generation, plants produce many antioxidant enzymes such as superoxide dismutase (SOD), catalase (CAT), ascorbate peroxidase (APX), glutathione reductase, monodehydroascorbate reductase, guaicol peroxidase, and glutathione peroxidase [42,103,104]. Exogenous BR application increases the production of many antioxidant enzymes across plant species. Enhanced production of SOD and peroxidases were seen in heat-stressed rice seedlings with application of BR [105]. Similarly, BR application in wheat pre-and post-flowering stage resulted in an increased APX and SOD activity when exposed to heat stress [106]. Therefore, enhanced ROS scavenging induced by BR provide increased tolerance to oxidative stress induced by heat.

The secondary messenger Ca^2+^ is one of the first molecules that propagate signals when a plant is experiencing high temperatures [107]. Ca^2+^ is involved in many signaling pathways and is necessary for induction of transcription factors (TFs), HSPs, and osmoprotectants in response to heat stress [108,109,110]. Specifically, induction of HSPs is regulated by the binding of heat shock transcription factor (HSF) family members [111]. For example, the DNA-binding activity of HSF to promote HSP expression in maize is mediated by Ca^2+^ [112]. Beyond HSPs, Ca^2+^ also stimulates the production of the osmoprotectant GABA in response to heat stress [113]. Roles of BR in regulating the movement of small secondary messenger molecules, including Ca^2+^, also could mediate plant responses to heat [114,115]. BR is known to increase cytosolic Ca^2+^ concentration by binding to the BR receptor, which results in opening of Ca^2+^ ion channels in the plasma membrane [116]. A further connection between BR and Ca^2+^ was identified in BR-induced antioxidant enzyme production, typically stimulated by stresses. In maize, Ca^2+^ flux, detected through the Ca^2+^/Calmodulin -dependent protein kinase (ZmCCaMK), was necessary for exogenous BR to increase antioxidant enzyme activities. Further, levels of ZmCCaMK itself is dependent of BR, indicating a feed-forward loop for Ca^2+^ response [117]. Though direct connections between BR signaling and Ca^2+^ in terms of heat stress require additional study, it can be suggested that BR regulate Ca^2+^-dependent signaling during heat stress response. Overall, BR play diverse roles in heat stress tolerance, ranging from small molecule to whole plant effects. BR transform normal plant processes to defend against heat stress by amplifying levels of protective genes, proteins and chemicals.

The many effects of heat stress just described can also be impacted through genetic manipulation of BR synthesis and signaling, further confirming the promising effects of exogeneous BR in cereals. Overexpression of a BR synthesis gene in *Brassica napus* plants increased seed yield and tolerance to heat and drought stress [118]. Overexpression of BR receptors conferred drought tolerance by eliciting the accumulation of osmoprotectants [119] and improved heat tolerance and better photosynthesis activity due to increased chlorophyll content and membrane stability as [120]. Consistently, knock-down expression of BR receptor resulted in decreased tolerance to high temperature [121] and knockout mutants of a BR negative regulator showed enhanced tolerance to heat, cold, drought, and salt stresses as compared to non-transgenic plants [122]. Together these data support that BR have a role in conferring tolerance to heat stress that can be exploited.

## 5. BR as Agrochemicals

Considering the effects of heat stress on cereal yields and the role of BR in mitigating those negative effects, we can understand not only why and how BR can be used to improve crop yields but also the information we still need to gather. Field experiments show that exogenously applied BR can significantly increase the yields of various crop plants [123]. BR impact multiple components of grain yield in cereals with exogenous BR application in wheat and rice increasing grain number per spike and panicle, respectively, number of tillers, degree of spike fertility, and the ratio of grain yield to biomass (harvest index) [123,124]. The mechanisms by which yield components are improved are complex, and likely arise in part due to improved plant biomass. In wheat, exogenous BR application increases leaf area, fresh and dry weights, which may be traced to the BR application increasing CO_2_ fixation and RUBISCO activity [125].

The beneficial effects of BR are not only limited to optimal conditions but also impact how plants respond to stress in field studies. For example, treatment with BR increased rice biomass, grain number and grain weight under heat stress relative to untreated controls [126]. Manipulating BR protects cereal yields in the field by mitigating many negative effects of high temperatures. Treatment with BR can maintain rice and wheat yields under heat and combined heat and drought stress, respectively, in some circumstances even at levels similar to plants grown at optimal conditions [9,10,21]. In rice, BR application achieves heat tolerance by maintaining essential plant activities, including photosynthesis rate and stomatal conductance by protecting photosynthetic machinery and alleviating photoinhibition [126,127]. Exogenous application of BR has also been shown to accelerate heat acclimation and provide thermotolerance by increasing survival rate of wheat seedlings. Improved photosynthesis and reduced membrane injury were observed in BR treated heat acclimated plants compared to untreated plants. Correspondingly, BR treatment increased thousand seed weight in heat acclimatized plants [128]. Given these positive impacts, integrating BR in agricultural practices by external application may be leveraged to maintain yields during heat stress.

However, slow advancement in BR research for heat tolerance specifically might be due to variability seen in BR action across studies. The inconsistency in agronomic research results arises due to different modes of BR application (seed soak, watering, foliar spray, etc.), concentration of application, specific BR compound applied, and also the developmental stage of target plant when BR was applied (Table 1) [129]. Additionally, publication bias also results in reports of the positive benefits of BR (shown in Table 1) and less data about the negative impacts. Generally, BR work in a narrow range of concentrations with negative impacts on desirable agricultural characteristics outside of those ranges [9,10,21]. As seen in *A. thaliana*, tomato, wheat, and maize, BR can cause inhibition of root growth at higher concentrations [130,131,132,133,134]. In cereal crops, systematic research on the relationship between BR and heat stress tolerance is needed, given the lack of studies examining heat stress especially relative to other abiotic stresses (Table 1). Additionally, more information is needed about the duration of exogenous BR impact. Nishikawa and colleagues [45] applied BR to wheat and cucumber and detected BR on the seventh day, but whether BR show impacts beyond that timepoint is unclear. This is especially relevant when considering the timing and type of application by cereal growers, laying out crucial applied research goals.

Regardless of knowledge gaps, efforts have been made to integrate BR and their positive impacts into crop production. For example, in Belarus and Russia, the BR compound 2,4-epibrassinolide has been registered for agricultural use since 1992 and used in commercial production of barley, tomato, potato and other vegetables [154]. The use of BR for agricultural purposes has proven both practical and economical, as demonstrated in Japan, China and Russia with costs of using BR comparable to the costs of other growth regulators, depending on the source [155]. Importantly, toxicity experiments show that BR have low toxicity, and they do not have any immediate negative effects in mammals, aquatic organisms, soil microbiological processes, and plants [156]. BR also are predicted to have no to very little long-term toxicological effects on the environment [154].

## 6. Conclusions and Further Research

Climate change has increased temperatures, limiting crop production. Plants can adapt to these environmental changes, but current projections of climate change shows that rising temperatures may outpace the ability of plants to adapt successfully. Hence, we need external resources to aide thermotolerance of plants. The Green Revolution arose due to the productivity of dwarf crop varieties that required substantial levels of water and nitrogen [157], which changed the face of agriculture. However, now with new problems and limitations arising due to climate change, the Green Revolution varieties are becoming less sustainable, and hence we must improve our methods to make more resilient varieties for current times. For example, evidence shows that Green Revolution developed semi-dwarf cereal varieties carrying *Reduced Height* (*Rht*) alleles do not perform well in drought conditions with yields lower than taller plants [158,159,160]. Further fine-tuning of Green Revolution lines is needed to combat increasing frequencies of extreme climate events. Regulating BR levels via exogenous application may be a way to balance the resource requirements bred during the Green Revolution in dwarfed varieties with resilience of wild, heat and drought tolerant relatives.

We can utilize BR in agricultural practices to boost cereal productivity and also improve heat stress tolerance. Genes involved in BR signaling and synthesis can be targeted in cereal breeding programs and modified using genetic engineering to increase yields [161]. For example, overexpression of *ZmDWF4*, a biosynthesis gene, improved grain yield per ear, increased heterosis of combinations by increasing both seed number and seed weight, improved leaf photosynthetic ability, improved cell growth, cell division and nutrient assimilation in transgenic lines compared to non-transgenic plants [162]. BR influence plant responses to changing environments through regulation of growth, development, and nutrient allocation [163], acting to simultaneously amplify heat stress responses and maintain plant growth. However, more systematic research needs to be done to test the effects of exogenous BR on different cereal species at various concentrations in diverse environments to get a complete understanding of their activity. These experiments can provide valuable information to growers about which BR compound to use on which species and also determine the appropriate concentration and timing of application. As seen in Table 1, BR experiments with cereals under heat stress are minimal, limiting our understanding of how to utilize BR for specific agronomic needs when exposed to heat stress. New and efficient ways of improving yields along with combating heat stresses need to be developed, and BR may be one of them. Agronomic studies together with molecular studies will link phenotypic changes induced by BR with the underlying molecular causes. More research needs to be completed to understand the transport of exogenously applied BR and how long effects last. Further how exogenous BR influence the synthesis and movement of other phytohormones, especially under heat stress in cereals, is an area needing attention. Building this knowledge and integrating knowledge of BR genes in breeding programs may be a useful target, facilitating breeding and engineering to boost cereal crop yields and enhance performance under environmental stress.

## Figures and Tables

**Table 1:** Positive impacts of BR in cereals under optimal and stressed conditions.

Compound	Application Method	Dose	Species	Stress	Reference
24-epibrassinolide	Foliar spray	10^−2^, 10^−4^, 10^−6^, 10^−8^ µM	Maize	None	[135,136]
		10^−3^, 10^−2^, 0.1 µM	Rice	Heat	[126,127,137]
		10^−2^ µM	Rice	Heat	[105]
		0.026, 0.052, 0.078 μM	Wheat	Saline	[138]
		0.05 µM	Wheat	Heat	[128]
		0.1 µM	Wheat	Drought and Heat	[124]
		0.52 µM	Wheat	None	[139]
		0.52 µM	Rye	Cold	[140]
		1 µM	Wheat	None	[125]
		2, 3 µM	Sorghum	Osmotic and Saline Saline	[141,142]
	Seed soak	10^−5^, 10^−3^, 0.1 µM	Rice	Saline	[143]
		0.052, 0.104, 0.156 μM	Wheat	Saline	[144]
		2.08 µM	Wheat	None	[139]
		2, 3 µM	Sorghum	Osmotic	[141]
		3 µM	Rice	Saline	[145]
		10 µM	Maize	Saline	[146]
	Other	0.01, 0.52 µM	Barley	Heat	[147]
		0.4 µM	Wheat	None	[148]
		10 µM	Barley	None	[149]
		400 µM	Wheat	Drought	[150]
Castasterone analogue	Foliar spray	10^−2^, 10^−4^, 10^−6^, 10^−8^ µM	Maize	None	[135]
28-homobrassinolide	Foliar spray	0.02, 0.1 µM	Wheat	Drought	[99]
		0.5, 1, 2 µM	Rice	None	[123]
		1, 2 µM	Wheat	None	[123]
		2, 3 µM	Sorghum	Osmotic	[141]
		101, 202, 404 µM	Wheat	Saline	[151]
	Seed soak	10^−3^, 10^−2^, 1 µM	Maize	Saline	[152]
		0.02, 0.1 µM	Wheat	Drought	[99]
		0.1, 0.5, 1 μM	Barley	None	[153]
		2, 3 µM	Sorghum	Osmotic	[141]
		3 µM	Rice	Saline	[145]

## Data Availability

Not applicable.
